# 

**Published:** 1785

**Authors:** 


### CATALOGUE.

1. OBSERVATIONS on the Climate of Naples, Rome, Nice, &c. in a letter to to Sir George Baker, Bart. M. D. In which is inserted some advice to those who intend visiting those places in pursuit of health. By *Benjamin Pugh*, M. D. 8vo. *Robinson*, London, 1784.

2. A physical Inquiry into the Cause and Cure of Fevers. By *Garret Hussey*, M. D. Licentiate of the College of Dublin, and Physician to the Merchants-Quay Hospital, Dublin. 8vo. *Robinson*, London, 1784.

3. Dissertations relative to the Natural History of Animals and Vegetables. Translated from the Italian of the Abbé *Spallanzani*, Royal Professor of Natural History in the University of Pavia, Superintendant of the public Museum, and Fellow of various learned Societies. To which are added, two Letters from Mr. *Bonnet* to the Author; and, to each Volume of this Translation, an Appendix; the first containing a Paper written by Mr. *Hunter*, F. R. S. and the Experiments of Dr. *Stevens* on Digestion; the second, a Translation of a Memoir of Mr. *Demours*, and Mr. *Debraw*'s Paper on the Fecundation of Bees. 8vo. 2 vols. *Murray*, London, 1784.

4. A Manual of Materia Medica, containing a brief account of all the Simples directed in the London and Edinburgh Diſpenſatories, with their ſeveral Preparations and the principal Compoſitions into which they enter. By *J. Aikin*, M. D. 8vo. Yarmouth, 1785.

5. Reports of the Humane Society, inſtituted in the Year 1774, for the Recovery of Perſons apparently drowned; for the Years 1783 and 1784. 8vo. *Dodſley*, London, 1785.

6. Medical Practice; a new and complete Syſtem, ſelected from original manuſcript Lectures of the moſt eminent Medical Profeſſors, by the late *T. Potter*, Surgeon at North Shields, near Newcaſtle upon Tyne. 8vo. 2 vols. London, 1785.

7. The remarkable Effects of fixed Air in Mortifications of the Extremities. To which is added the Hiſtory of ſome Worm Caſes. By *John Harriſon*, Surgeon, of Epſom, Surry. 8vo. *Bladon*, London, 1785.

8. Obſervations on the Properties and Effects of Coffee. By *Benjamin Moſely*, M. D. 8vo. *Stockdale*, London, 1785.

9. A Treatiſe on the Mineral Waters of Balaruc, in Languedoc, in the South of France; containing their origin and diſcovery; nature and analyſis; their properties, and the manner of uſing them; with Obſervations on the wonderful Cures recently performed by them, particularly in paralytic cafes. By *M. Pouzaire*, M. D. of the Faculty of Montpellier, refident at the Baths of Balaruc. Printed in French, and tranflated into Englifh, by *Benjamin Pugh*, M. D. With fome additional Cafes under his own infpection; and fome Remarks on the City of Montpellier, its Environs, Univerfity, and the different Routes from England to the faid Baths. 8vo. Chelmsford, 1785.

10. Thoughts on the Formation and Properties of the different Kinds of Air. With Remarks on Vegetation, Pyrophori, Heat, cauftic Salts, Mercury, and on the different Kinds of Air. 8vo. *Faulder*, London, 1785.

11. An Effay on the Retroverfion of the Uterus; illuftrated with Cafes and Obfervations. By *William Cockell*, M. D. of Pontefract. 4to. *Law*, London, 1785.

12. Letters on the Elements of Botany, addreffed to a Young Lady, by the celebrated J. J. Rouffeau. Tranflated into Englifh, with Notes, and twenty-four additional Letters, fully explaining the Syftem of Linnæus. By *Thomas Martyn*, B. D. 8vo. *White*, London, 1785.

13. A Medical Commentary on fixed Air. By *Matthew Dobfon*, M. D. F. R. S. The fecond

ſond Edition; with an Appendix on the Uſe of the Solution of fixed Alkaline Salts, ſaturated with fixable Air, in the Stone and Gravel. By *William Falconer*, M. D. F. R. S. and Phyſician to the General Hoſpital at Bath. 8vo. *Cadell*, London, 1785.

14. *Richardi Relhan*, A. M. Collegii Regalis Capellani, Flora Cantabrigienſis, exhibens plantas agro Cantabrigienſi indigenas, ſecundum ſyſtema ſexuale digeſtas; cum characteribus genericis, diagnoſi ſpecierum, ſynonymis ſelectis, nominibus trivialibus, loco natali, tempore infloreſcentiæ. 8vo. Cantabrigiæ, 1785.

15. *Jacobi Dickſon* Faſciculus plantarum cryptogamicarum Britanniæ quæ in Floris Hudſoni, Lightfootii, et Curtſii, non reperiuntur, Tabulis aeneis illuſtratus. 4to. *Nicol*, London, 1785.

16. Reflections on the Study of Nature, tranſlated from the Latin of the celebrated *Linnæus*. 8vo. *Nicol*, London, 1785.

17. Afhandling om Huſhoallningen til ſjoes &c.; *i. e.* A Treatiſe on the Management of Ships and the Health of ſea-faring People. By *Arvid Faxe*, M. D. Phyſician to the Admiralty. 8vo. Carlſſcroon, 1782.

18. Die Geſchichte der Kriebelkrankheit &c. *i. e.* Hiſtory of the *Necroſis Uſtilaginea* of Sauvages,

Sauvages, particularly of that Species of it which prevailed in the Neighbourhood of Zell in 1770 and 1771. By *J. Taube*, M. D. Physician to the Court, and Member of the Royal Œconomical Society at Zell. 8vo. Gottingen, 1782.

19. Praktische und vortheile und verbesserungen verschiedener pharmaceutisch-chemischer Operationen &c.; *i. e.* Pharmaceutico-chemical Processes improved and rendered more advantageous for Apothecaries. By *J. F. A. Goettling*. 8vo. Weimar, 1783.

20. Geschichte der Medinischen und Physikalischen elektricitæt &c.; *i. e.* History of Medical and Physical Electricity, and of the most recent Experiments in that Science; compiled from the latest Works, and augmented with Experiments peculiar to the Author. By C. G. *Kuhn*. Part I. 8vo. Leipsic, 1784.

21. Acta Medicorum Suecicorum sive Sylloge Observationum et Casuum rariorum in variis Medicinæ partibus, præsertim in Historia Naturali, praxi Medica et Chirurgica. Tom. I. 8vo. Upsal. 1783.

22. Collectio opusculorum selectorum ad Medicinam forensem spectantium &c. Cura

 *Joh.*

*Joh. Chrift. Traugolt Schlegel*, M. D. 8vo. Leipfic, 1783.

23. Obfervationes de febre petechiali. Auctore *Lud. Chrift. Althof de Detmold*, Med. et Chir. Doct. 8vo. Gottingæ, 1784.

24. Differtatio Inauguralis de vera Diabetis Caufa in defectu affimilationis quærenda. Auctore *Franc. Place*, Eboracenfi. 4to. Gottingæ, 1784.

25. De defpiciendis Artium et Medicinæ irriforibus, differitur *Carol. Gebler*, M. D. Anatom. et Chirurg. Prof. Publ. Ordin. &c. 4to. Lipfiæ, 1784.

26. Differtatio Medica de acrimonia Urinofa in corpore humano retenta. Auctore *Simon Neuburg*, Med. & Chir. Doct. 4to. Gottingæ, 1783.

27. Differtatio Medica de aeris fixi ufu medico nuper celebrato. Auctore *Carolo Johan. Nyberg*. 4to. Jena, 1783.

28. Tractatio de quibufdam notabilioribus objectis ad artem obftetricandi fpectantibus, tironum ufui deftinata. Auctore *Chrift. Jacob. Theophilo de Meza*, M. D. 8vo. Havniæ, 1783.

29. *Jacobi Henrici Proepffer* Differtatio de Caufis Phthifeos Pulmonalis. 8vo. Jena, 1784.

30. *Chrift.*

30. *Chrift. Henric. Krumbholz* Differtatio Medica fiftens examen feminis mulieris. 4to. Jena, 1784.

31. Differtatio de virtute Boracis medicinali dubia. Auctore *Joanne Freder. Meticke.* 4to. Jena, 1784.

32. Differtatio Medica fiftens momenta quædam de Mofcho naturali et arte facto. Auctore *Gab. Gottlieb Reinick.* 4to. Jena, 1784.

33. Differtatio Medica Zincum Chemicum inquirens. Auctore *Henr. Emmanuel Geller.* 4to. Jena, 1784.

34. Defcriptio Anatomica nervi cruralis et obturatorii, icone illuftrata. Auctore *Martino Ernefto Styx.* 4to. Jena, 1784.

35. Differtatio Medica de ufu Dulcamaræ. Auctore *Johanne Godofredo Otto.* 4to. Jena, 1784.

36. Obras Pofthumas de D. *Andre Piquer,* Medico, &c.; *i. e.* The Pofthumous Works of Don *Andrew Piquer,* Phyfician, &c. With the Life of the Author, by his Son, D. *Juan C. Piquer,* Prieft, and Chaplain to His Majefty in the Royal Monaftery of the Vifitation. 8vo. Madrid, 1785.

